# With good intentions: complexity in unsolicited informal support for Aboriginal and Torres Strait Islander peoples. A qualitative study

**DOI:** 10.1186/1471-2458-11-686

**Published:** 2011-09-04

**Authors:** Nathaniel J Ward, Tanisha Jowsey, Penny J Haora, Clive Aspin, Laurann E Yen

**Affiliations:** 1 Australian Primary Health Care Research Institute, Australian National University, Ian Potter house, Cnr Marcus Clarke and Gordon streets, Acton 0200 Canberra, Australia; 2 Menzies Centre for Health Policy, Australian National University, John Curtin School of Medical Research, Garran Road, Acton 0200 Canberra, Australia; 3 Poche Centre for Health Research, University of Sydney, Sydney Medical School, Edward Ford Building A27, 2006 NSW, Australia

**Keywords:** Aboriginal, Indigenous, chronic heart failure, chronic illness, chronic obstructive pulmonary disease, diabetes, qualitative methods, self-management

## Abstract

**Background:**

Understanding people's social lived experiences of chronic illness is fundamental to improving health service delivery and health outcomes, particularly in relation to self-management activity. In explorations of social lived experiences this paper uncovers the ways in which Aboriginal and Torres Strait Islander people with chronic illness experience informal unsolicited support from peers and family members.

**Methods:**

Nineteen Aboriginal and Torres Islander participants were interviewed in the Serious and Continuing Illness Policy and Practice Study (SCIPPS). Participants were people with Type 2 diabetes (N = 17), chronic obstructive pulmonary disease (N = 3) and/or chronic heart failure (N = 11) and family carers (N = 3). Participants were asked to describe their experience of having or caring for someone with chronic illness. Content and thematic analysis of in-depth semi-structured interviews was undertaken, assisted by QSR Nvivo8 software.

**Results:**

Participants reported receiving several forms of unsolicited support, including encouragement, practical suggestions for managing, nagging, growling, and surveillance. Additionally, participants had engaged in 'yarning', creating a 'yarn' space, the function of which was distinguished as another important form of unsolicited support. The implications of recognising these various support forms are discussed in relation to responses to unsolicited support as well as the needs of family carers in providing effective informal support.

**Conclusions:**

Certain locations of responsibility are anxiety producing. Family carers must be supported in appropriate education so that they can provide both solicited and unsolicited support in effective ways. Such educational support would have the added benefit of helping to reduce carer anxieties about caring roles and responsibilities. Mainstream health services would benefit from fostering environments that encourage informal interactions that facilitate learning and support in a relaxed atmosphere.

## Background

As global chronic illness prevalence and burden have increased during the past 15 years so too has the body of research evidence identifying the absolute necessity to engage people with chronic illness in self-management. Increasing people's capacity and motivation to engage in self-management behaviour has been a widely used strategy to improve their health outcomes and address the increasing pressures on health systems [1-4]. Australia has been no exception to these trends; the prevalence and burden of chronic illnesses in Australia is high, as is health policy attention to improving chronic illness management [5,6] through increased self management activity.

Currently primary health care services utilise strategies such as motivational interviewing to increase people's knowledge and activation in self-management behaviour. Health services also have access to a great raft of self management education tools [4,7,8]. However, little formal support is provided to carers and family members even though they are known to influence patient self-management [9]. In the case of Aboriginal and Torres Strait Islander peoples, who are Australia's indigenous people, the influence of family members and community on people's experiences of chronic illness may be even more pronounced due to both the higher prevalence of chronic disease in the community and the nature of kinship obligations.

Questions about how informal support is experienced by people living with chronic illness are integral to discussions of improving and promoting better self-management strategies. In her critical discussion of some modes of engaging people with chronic illness, Greenhalgh comments that "Most conventional chronic disease self-management programmes and policies take a biomedical view of the self, characterised by capacity and motivation to perform certain goal orientated tasks expected by doctors and nurses" [[10]: 630]. Speaking also to this model Finkler writes: "This (biomedical) model conceives of the person as an autonomous unit, independent of and isolated from other individuals and the social and cultural contexts. By not incorporating information about the family and the life world in which the patient is embed, the medical consultation aggravates rather than allays the crisis for the patient" [[11]:126]. Unpacking and understanding the ways that wider social and cultural contexts impact on people with chronic illness, therefore, is integral to formulating better health interventions aimed at increasing engagement with self-management behaviours and thereby ultimately improving health outcomes [12-14].

Drawing on constructivist approaches to understanding the experience of chronic illness Martin and Peterson suggest that such experiences are at one and the same time social as well as medical. Indeed they suggest that the medical is equally part of the *social *experience of illness [15]. The person living with a chronic illness in this regard draws in those who are within their peer and family network, all of whom do the 'main work' of managing a chronic illness. "Such 'work of managing'," write Martin and Peterson, "can, and often does, involve the lives and identities of the chronically ill and their families in multiple domains financial, emotional, sexual, economic, social and vocational."[15: 580] Understanding the dynamics and interplays of these contexts is especially important, since with multiple players and domains there are complexities in the context and the ways that meanings will be located by each actor [16].

The Serious and Continuing Illness Policy and Practice Study (SCIPPS) aimed to develop policy and health system interventions that are patient-centred and support the provision of optimal care for people with chronic illness and carers of family members. To address this aim SCIPPS began with qualitative research, undertaking nineteen semi-structured interviews with Aboriginal and Torres Strait Islander people, which explored their experiences of living with chronic illness. We found that family members and the wider community played an integral role. In particular, they provided both solicited and unsolicited informal support. This paper addresses the question: How do Aboriginal and Torres Strait Islander people with chronic illness experience informal unsolicited support?

In part, understanding the complexity of context around unsolicited support is in recognising that such support is not always what we could call an unmitigated good. That is to say, while it may be helpful in some ways, it may have consequences that create tensions and ambiguities in relationships and self-management practices. There are resonances here with what Broom discusses as 'latent functions' or 'unintended consequences' in connection with preventative health interventions [17]. Such unintended consequences might include stigmatisation of 'sick' people, where the fact of being ill becomes a personal moral failing, or where the experience of feeling forced to follow a regimen may actually result in a kind of rebellious non-compliance as a reassertion of personal and adult autonomy [17] [see also [18,19]]. Successes in health outcomes may still arise, however finely nuanced complications may arise as *emergent *properties of the support context. As we will suggest later, and in relation to the experience of the Indigenous participants that inform what is presented here, certain tensions, ambivalences, and anxieties can arise as unintended by-products of flows of informal support within family and peer networks.

It is within this frame of reference that the findings of this paper describe the way people with chronic illness are offered support by family members for engaging in self-management behaviour and coping with experiences of chronic illness more generally. In this way, our concerns articulate with the notion that understanding people's social lived experiences of chronic illness is fundamental to improving health service delivery, particularly in relation to self-management activity. As such, we argue that social lived experience, including forms of informal support, must be factored into the landscape of health service interventions.

## Methods

SCIPPS focused on three serious and long-term diseases-complicated type 2 diabetes ('diabetes'), chronic obstructive pulmonary disease (COPD) and chronic heart failure (CHF)-which were found to be common, costly and require ongoing care from multiple providers and services. The SCIPPS qualitative study involved semi-structured interviews with 61 participants who had DM, COPD and/or CHF, and 17 family carers, as well as focus groups with 66 health professionals who care for people with these conditions. This paper reports key findings from the analysis of 19 interviews with Aboriginal and Torres Strait Islander participants who had chronic illness (N = 16) or cared for a family member with chronic illness (N = 3).

### Recruitment

Data collection occurred between March 2007 and November 2009. Nineteen Indigenous participants with DM (N = 17), COPD (N = 3) and CHF (N = 11) were recruited by purposeful sampling through referrals from Aboriginal Medical Services (AMS) and general practices in Western Sydney in New South Wales and the Australian Capital Territory. Eligible participants included people with one or more of these three conditions aged between 30 and 85 years. The relatively low age cut-off was chosen to reflect the earlier onset of chronic illness and shorter life expectancy of Indigenous Australians [20]. Those included range in age from 34 to 70 years. Percival (2004) and Wagner (1998) argue that family carers of people with chronic illness can provide important insight into the experiences of people living with chronic illness [21,22]. Three family carers of people with the sentinel chronic illnesses (two were married to participants with chronic illness) were recruited through an Indigenous informal chronic illness support group. Participants were not randomly selected, and as such are not representative of the health service population, nor the Aboriginal population overall.

The data collection and analysis was guided by Lincoln & Guba in terms of the credibility, confirmability, transferability, and dependability to maximise the rigour of the study [23]. We followed the advice of staff of the recruiting AMSs as well as members of the Indigenous Health Interest Group of the Australian National University to ensure appropriate Indigenous health research methods and community engagement [24]. Interviews continued until saturation of themes occurred [25] at which point the dataset was closed and completed with 19 participants.

### Interviews

Semi-structured in-depth interviews were conducted with participants by four researchers with experience in multi-cultural research (none of whom were Indigenous to Australia). Each interview ran for between 45 and 90 minutes, following a semi-structured interview guide. People with chronic illness and carers also completed a 10 minute demographic survey and provided information about their health conditions and health care encounters. Participants were asked to describe their experience of living with a chronic illness. The research team judged that sufficient data had been gathered when interviews were no longer providing new insights or ideas central to the experience of having DM/COPD/CHF, indicating data saturation.

Study approval was obtained from the Australian National University Human Research Ethics Committee, the ACT Health ACT Human Research Ethics Committee, the University of Sydney Human Research Ethics Committee, Sydney West Area Health Service Human Research Ethics Committee and the Aboriginal Health and Medical Research Council of NSW.

Consent was obtained from all participants prior to interview. The data collection and analysis were carried out by a group of six researchers with backgrounds in health and social sciences.

The sub-study of Aboriginal and Torres Strait Islander experiences was planned with and informed by our ACCHS/AMS collaborators; who advised that participant confidentiality was of utmost importance (particularly given the small sample) and accordingly as much identifying data as possible has been removed from this paper and the reference 'participant A, B..' is used, as advised by the ACCHS/AMS collaborators. The ACCHS/AMS collaborators approved this paper prior to its submission for publication.

### Analysis

All interviews were electronically recorded and transcribed verbatim. The data were analysed using qualitative content and thematic analysis, assisted by QSR NVivo8 [26]. The data from participants with chronic illness was analysed together with family carer data and these datasets were also analysed separately. The research team modified, by iteration, the coding scheme used in the original qualitative study (Jeon [13]). This scheme was used to code all transcripts (each transcript was coded by three members of the research team and checked by two team members to ensure rigour). Following Morse & Field, we used content analysis to identify issues in the data which were commonly raised by participants [25]. The content analysis was assisted by frequency matrix coding in NVivo8. These issues were then further explored thematically. Descriptive analysis (frequencies, means, modes and medians) of the survey data was undertaken using SPSS version 15 [27].

## Results

Participants reported different dimensions of informal support. Informal support can be understood as that type of support that occurs outside of health service interfaces. While there are ways that participants discussed solicited support--that is by directly or indirectly asking for help in some way--our concern in what follows is in tracing participants' representations of *unsolicited *informal support. Unsolicited support, then, is not directly or explicitly sought by participants. It includes instances of experiencing encouragement and practical suggestions for managing, but is not necessarily limited to these things. It also includes experiences of 'nagging', 'growling', and 'surveillance'. It is important to signal here that people may not always automatically assist a chronically ill person of their own accord, nor will people with chronic illness necessarily believe that such support will be provided.

Although the findings presented below are drawn from Indigenous participants, and in this sense some of them carry cultural inflections unique to that setting, many of the experiences are not necessarily particular to the fact of indigeneity [see for example [14]].

### Unsolicited psychological support: complexities of the 'yarn'

In discussions of receiving peer support there was an emphasis on the value of getting together and having a 'yarn', of talking about things; often with others who have experience of living with chronic illness. The value of the 'yarn' is something that is saturated with ideas about having a laugh, of getting together and feeling like everything is alright. In addition, certain spaces such as friend's houses and AMS waiting rooms were articulated as being naturally conducive to 'yarning' and can thus be interpreted as 'yarn' spaces. One participant with DM and CHF who regularly met with a friend who also had DM put it this way: *"I go up to my mate's place, and you know, have a yarn about this and [that] ... and ah have a bit of a laugh about it, you know? Everything's right, you know? Back to normal" (Participant_A)*. In this way the value of the 'yarn' is framed in terms of its ability to put the person with chronic illness at ease, to relax them. Another participant accented it differently in response to how he thought being Aboriginal helped him manage his conditions:

"I couldn't say for sure ... but, we share a lot. You know when we meet people we talk about things. It's like when you go in to a medical centre, you as a whiter person, you might be lucky if somebody says hello to you. If we go in and I know someone we'll have a good yarn. How you going with yours? You got diabetes yeah. How many tablets are you on? I'm on the needle. "Why? How high does yours get?" So there's always that yarn that we can pass on that information, "What do you do about it?" and all this stuff, and I think sharing a lot of the things that we do that's the difference because we're so small in numbers even though we've got the largest population in the country in this area I think we can walk in anywhere and we always get a "G'day" and a yarn. And not only that, if you're a bit nervous then it calms you down, a lot of us so there's a lot of aspects I suppose we think on a cultural basis." (Participant_B)

The emphasis on sharing through 'yarning' has important contextual threads. There is, for example, its deployment in responses around marking something unique in Aboriginal and Torres Strait Islander peoples' styles of relationship. Beyond this, it is deployed as a way of mitigating fear or anxiety as a component of the lived experience of chronic illness. But there is a further contextual complication here, for while 'yarning' with peers or family provides a form of informal psychological support, the wider realities of being surrounded by others who are sick can be anxiety producing in and of itself. The following participant draws awareness to the tension in this way:

*"Sometimes, you get someone to talk to, you've always got someone to talk to, you know you run into someone you've got relatives over here and friends over here, people have been through it you've known for years, and you think oh geez I'm going to go that way, I know a woman that's had it she's had 3 or 4 heart attacks, 3 or 4 operations you know and she's on a dialysis there and I'm thinking you know I wonder if I'm going to be on that thing one day, you know. So there is people that are around that you see around that have more or less got the same thing, the same problem. Yeah you just talk to them see how they cope with it and then you try and cope with it as best you can." (Participant_C)*.

So while the value of community, family and 'yarning' is a significant source of support and knowledge, it is also significant for the fact that it drives some forms of ambivalence in experiences of chronic illness.

### Unsolicited practical support

In addition to what we have framed as unsolicited psychological support, participants also conveyed instances of unsolicited practical support. One such case of unsolicited practical support comes in response to the question of what kind of role, if any, a participant's family played in providing support. He commented:

*"Well yes. in a way you know. There's been times where they've given support like driving or maybe some dosh for some extra expenses or if I run out of food for the fortnight and they might help, I don't have to ask, they come and have a look and first thing you know kids do whether they're grown up or not, they open the fridge they say, 'Oh well we'll go down and get you a few of these.' I say 'Ooh.' When you get there they say, 'Well what else do you want?' I never say, this, this and this. 'Come on you must need something' so. But they're quite good in that way, support today" (Participant_B)*.

This case has several interesting dimensions. Earlier in his interview the participant placed a great emphasis on not getting his family involved directly in the management of his condition, stating that his independence in this regard was important to him. That is, he was not given to overtly soliciting support from his children. Nevertheless, the family provided him unsolicited practical support by opening the fridge and assessing its contents, something they used to do as young children to take food out. In an inversion of this history they go to the fridge to see what needs to be 'put in', mobilising a shared memory of provision and family life in the process. There are two consequences of this, the first is that by trading on this memory they are able to offer support in a way that does not impinge on their father's sense of independence. The second is that the support is normalised through the choice of deployment around notions of reciprocal care spanning the life-cycle and processes of daily family living.

### Unsolicited psychological and practical support

At times unsolicited psychological and practical support provided by family, friends and the community was combined. A man in his thirties with DM described his experience of binge drinking in response to his diagnosis of DM. In the following example he draws attention to his response to a friend's suggestion that his binge drinking was out of control;*"And it was like; this has come from a heavy drinking Cook Islander. And I went, 'what do you mean mate'? He said 'well, look,' he says, 'I know we come down here, but you're not the same person'. You know? And that was when I decided to get help" (Participant_D)*. This comment on his alcohol-related change in personality is a kind of support that is more subtle than just being either psychological or practical. Instead, the drawing of his attention to the binge drinking caused what can be termed a 'reflexive turn' in the participant, one that ultimately ended in his seeking help.

### Responses to support

Acts of unsolicited support were often expressed in terms of gratitude for having access to support and encouragement from family, "*if you haven't got family"*, one participant remarked, *"I don't know how the damn hell they do it" *(*Participant_B*). However, there were also expressions of ambivalence toward 'messages' conveyed through support, especially when they presented in the form of nagging, growling and surveillance. For example, a participant said "*my nieces and that they all have a growl at me*" *(Participant_E)*. When asked how her family helped with her diabetes another participant responded, "*Well just by nagging me, and saying to me all the time... 'you shouldn't be having that Mum' or 'should you be having that?'. Yeah so ... Mary she's the worst one*" *(Participant_F)*. There are hints from this same participant that this form of support does not mitigate the personal difficulties of managing a condition, serving rather to foster feelings of ambivalence:

*"I mean I love to have a drink of Coke occasionally you know, and Lucy says to me 'Mum, you could, try the... try the Zero' .... So I thought oh and I bought a bottle and she said 'I'm really proud of you Mum' she said 'that you've bought that' she said 'have that instead of...' I said 'yeah I know but...' so I don't know it is difficult." (Participant_F)*.

The ambivalence surfacing around difficulty centres on a particular tension. This tension arises in the interstices of wanting positive reinforcement from family and understanding that the family cares, juxtaposed against feeling a disruption to her biography [28] through being thwarted insofar as she cannot engage freely in a lifestyle she might choose for herself:

*"You know I'll always think 'oh well what's the... what's the difference' you know, might as well die happy as... I'm happy, struggling but... yeah. And it's the wrong attitude I'm afraid to have because I... because my girls said 'what about your grandkids?' That's what they say to me." (Participant_F)*.

What also comes out quite strongly in this example is the way that the 'nagging' as a form of unsolicited support references social surveillance and trades on perpetrating a sense of guilt. While this is deployed around notions of the participant being important to the grandchildren and family, and in this sense can be read as connecting to notions of a caring family, it also draws on notions of responsibility. The responsibility in this regard is a responsibility to live healthily, and by extension to live for the family. This notion negates the idea that the death of grandparents is something that is normal as part of the life course. Similarly, as part of the construction of living with chronic illness, it makes the chronically ill person directly responsible for staying alive, rather than this being something that is out of their control. It is not just a matter of how to live, but rather of how long to live, even at the expense of sacrificing some personal happiness.

### The carer perspective

A woman who cared for her partner with DM reported trading on notions of responsibility in a different way. She attempted to provide unsolicited support for him by using a sign language that was common between them but could be hidden from others. When he ate food with other members of the community that she knew would make him feel unwell she would *"give him the look, that serious look of 'that's enough'" (Participant_G)*. This approach was met with limited success in that its hidden or silent nature meant it could be easily ignored. She went on to say:

*"So it's quite emotionally exhausting for me when I sit there and I watch it and I know that later he's going to suffer, it's really quite tiring. And how do I... I need to be respectful of culture and his ways but at the same time I can't afford to have a man who's sick. So then generally when people leave within the hour we've got the BSL, we've got the glucometer out and we've got the blood sugar thing happening... So it's very tiring" (Participant_G)*.

In reports of support from our three carer participants, it was often impossible to gauge whether the support was explicitly solicited or otherwise, but for the most part their reports seemed to stem from a common understanding between their care recipient and themselves that support would be provided (although this may not have been voiced), even when they did not know exactly how to provide support. A woman who cared for her husband described an instance where she provided support that she first framed as helping the nurses and then added that in doing so provided unsolicited support for her husband:

*"you do do that, you just go ahead and you say 'Look, I'll shower him while I'm here' and I think that the nurses really appreciate that too, because sometimes they say, 'Oh no, it's alright, we'll get there' and I'll say 'No, I'm here, I'll do him' and you know, that was okay, and give him his clothes to make him feel a bit better, because it's such a long time when they're so sick" (Participant_H)*.

She also described her experience of not feeling confident in her knowledge to help manage his home dialysis; *"I really didn't know what I was doing actually, to tell you the truth. And I used to have to just sort of gauge everything and be, 'oh, I hope I'm doing it right', you know" (Participant_H)*. In describing her overall experience of caring for her husband she signalled the exhausting and demanding nature of undertaking a heavy load of caring responsibilities; however, she asserted that taking any kind of break from providing the increasing home care needs of her husband was something she would not do despite her exhaustion; *"you couldn't do that, because when you've got someone who is so ill and you know that they need you, you can't. You just can't go and have that break" (Participant_H)*.

## Discussion

The participants in this study -- all of whom were experiencing chronic illness either directly or indirectly -- experienced support from or provided support for family members. In the case of those participants who had a chronic illness, the experience of support also extended to that received from peers or community members. Their responses to this support varied from a deep sense of gratitude to ambivalence and anxiety. The experiences of the three family carer participants provided valuable insights into the complex and exhausting task of providing informal support for family members who have chronic illness. It is important in the following discussion of unsolicited informal support not to lose sight of the efforts and needs of family carers who provide such support. Indeed, family carers provide important contributions to socialising the entrenched medicalisation of chronic illness [15]. Similarly, the roles carers play speaks to the complexity of the overall terrain in which flows of support operate and interface with formal health service environments (evidenced by the carer participant who helped the nurses whilst also helping her husband, for example). The high demands of care on informal carers and the 'costs' of caring they encounter [29] are not currently matched by sufficient formal support from health policy and services [9]. This issue is not limited to the Indigenous context. There is growing recognition by practitioners and policy makers that illness in individual is not seen by Aboriginal and Torres Strait Islander people as the problem of the individual, but of the family and community, which makes the need to provide support to informal carers within that context crucial to good health outcomes.

In terms of our overall findings, there are indications that certain locations of responsibility in informal support are anxiety producing for people with chronic illness and their family carers, as with the examples of 'nagging' and 'growling', as well as those that involved engaging in and acting on surveillance. While such unsolicited informal support demonstrates notions of care, its inherent tension can be counterproductive. In relation to our findings we have discussed this in terms of an ambivalence that arises as a result, and which is in this way one of the unintended consequences or emergent properties of the support dynamics. Indeed, this type of support seems to connect with what Shigaki and colleagues describe as 'controlled motivation'[30]. In their study, which draws on self-determination theory, they found that types of controlled motivation (arising in response to nagging and surveillance, and other potentially guilt-inducing behaviours) in no way predicted any improvements in individuals' motivations to engage in effective self management behaviour. Nevertheless, the qualitative material we have does suggest it is sometimes effective for motivating people to self-manage while at the same time fostering feelings of both ambivalence and anxiety. In this sense, it can have negative emotional effects. Such effects might have flow on consequences in terms of people's psychological health [cf [31]].

While responsibly managing a chronic condition is important, constant reminders about management runs the risk of directing a person towards coming to feel like they are their illness. It is in this way that the value of having a 'yarn' becomes apparent, as it creates a chance for people to 'feel normal.' But there is a tension here too, as our results show that even with 'yarning' other anxieties can arise around whether or not one will suffer the same fate as one's peers. The question becomes one of how to cultivate support practices that encourage people to feel 'normal' [19] but also mitigate anxieties around reflecting on the nature of being sick and what outcomes may occur as a result. Cultivating practices of support that encourage this should form part of concerted education and training efforts aimed at giving family members effective tools for providing support. Family carers must be supported with appropriate education so that they can provide support, both solicited and unsolicited, in an effective way. This is consistent with the recommendations made by Rosland et al, who make the twofold point that "Future interventions should help patients with chronic illness overcome barriers to self-care, and help families support these patients in ways that patients will perceive as positive and will effectively improve patient outcomes" [30]. This suggestion also has resonances with the findings of Martire et al in relation to people with osteoarthritis. They suggest that education interventions that incorporate the spouse or partner of a chronically ill person showed that "patients ... experienced greater improvements in spousal support and punishing responses [such as anger and irritation] than those who received support without spousal involvement" [31: 191]. An important risk to acknowledge in our assertion that more support is needed for family carers is that our increasing funding for and reliance on family carers actually increases social surveillance of people with chronic illness [17]. With this in mind, it is critical that future family carer education interventions actively take steps to minimise anxiety-producing behaviour and moderate surveillance. Future support initiatives must also factor in the emotionally and physically exhausting nature of the caring role and empower family carers in order to mitigate this.

### Limitations

We did not aim for generalisability; rather, we aimed for a small representative sample of people with the three index conditions, saturation of issues raised in responses from our participants, and coherent interpretations of our data. While the research was conducted across two local sites the findings do not indicate they are site-specific.

## Conclusions

Australian health policy has signalled an increasing shift toward reliance on individuals and families to engage in self-management behaviour in relation to chronic illnesses. This shift has not been equally accompanied by critical analysis of the implications for people with chronic illness and informal family carers. Nor has there been sufficient education and interventions for increasing family members' capacity to provide appropriate unsolicited support. Government and health care service attention to these matters is essential.

## Competing interests

The authors declare no competing interests. The funding organisation (NHMRC) had no role in the study design, data collection, analysis and interpretation, or the writing and publication of this article.

## Authors' contributions

NJW coded data, undertook primary analysis of the findings, contributed to primary analysis of the findings, and contributed to drafting and critical revision of the manuscript. TJ participated in design and conduct of the study, coded data and checked coding, contributed to primary analysis of the findings, and contributed to drafting, critical revision and editing of the manuscript. LY participated in design and conduct of the study, and contributed to critical revision of the manuscript. CA participated in design and conduct of the study, checked coding, and contributed to critical revision of the manuscript. PH coded data and contributed to critical revision and editing of the manuscript. All authors read and approved the final version of the manuscript.

## Pre-publication history

The pre-publication history for this paper can be accessed here:

http://www.biomedcentral.com/1471-2458/11/686/prepub
